# Targeted Protein Degradation by Chimeric Small Molecules, PROTACs and SNIPERs

**DOI:** 10.3389/fchem.2019.00849

**Published:** 2019-12-10

**Authors:** Mikihiko Naito, Nobumichi Ohoka, Norihito Shibata, Yoshinori Tsukumo

**Affiliations:** Laboratory Molecular Target and Gene Therapy Products, National Institute of Health Sciences, Kawasaki, Japan

**Keywords:** PROTAC, SNIPER, E3 modulator, ubiquitin, proteasome, protein degradation

## Abstract

Technologies that induce targeted protein degradation by small molecules have been developed recently. Chimeric small molecules such as Proteolysis Targeting Chimeras (PROTACs) and Specific and Non-genetic IAP-dependent Protein Erasers (SNIPERs), and E3 modulators such as thalidomides, hijack the cellular machinery for ubiquitylation, and the ubiquitylated proteins are subjected to proteasomal degradation. This has motivated drug development in industry and academia because “undruggable targets” can now be degraded by targeted protein degradation.

## Modalities of Recent Drug Development

Development of a therapeutic antibody and a small molecule inhibitor is the most successful strategy to develop novel molecular target drugs these days (Nelson et al., 2010; Ferguson and Gray, 2018). The targets for antibodies include tumor specific antigens such as human epidermal growth factor receptor 2 (HER2) expressed on breast cancer cells that is recognized by Trastuzumab, and immune suppressive molecules such as programmed death-1 (PD-1) and programmed death-ligand 1 (PD-L1) recognized by Nivolmab and Pembrolizumab, respectively. However, antibodies cannot penetrate into cells, and therefore, target molecules for antibodies are limited to cell surface and extracellular proteins. In contrast, small molecule inhibitors can penetrate into cells and effectively inhibit the function of target proteins, such as kinases and proteases. However, developing small molecule inhibitors against proteins that do not possess enzymatic activity is challenging. Therefore, many intracellular proteins without enzymatic activity are unable to be targeted by antibodies and small molecule inhibitors, and they are sometimes called “undruggable targets.” These include scaffold proteins, transcription factors and splicing factors, and account for more than 70% of the proteins expressed in cells.

Accumulating evidence suggests that inducing protein degradation by small molecules represents a promising approach to make “undruggable targets” druggable. There are reports that small molecules, thalidomides and sulfonamides, induce the degradation of “undruggable targets” such as transcription factors (Ikaros and Aiolos) (Krönke et al., 2014; Lu et al., 2014) and a splicing factor (RBM39/CAPERα) (Han et al., 2017; Uehara et al., 2017). Technologies to induce protein degradation by chimeric molecules, Proteolysis Targeting Chimeras (PROTACs) and Specific and Non-genetic IAP-dependent Protein Erasers (SNIPERs), have been developed, which enables rational design of degrader molecules against target proteins of interest. This mini-review provides an overview of the protein degradation technologies.

## Classification of Degrader Molecules

**Figure 1:**
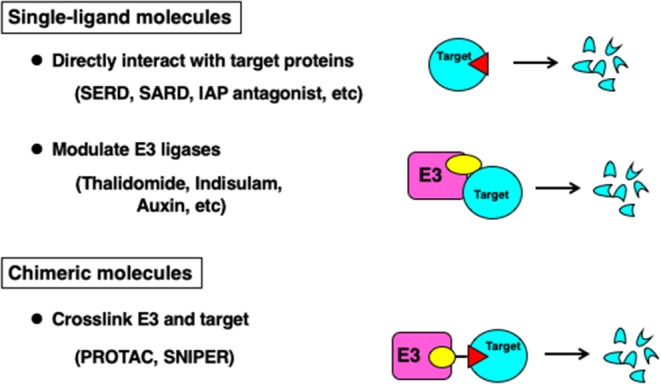
Classification of degrader molecules.

Small molecules that induce degradation of target proteins can be classified into three groups depending on the structure of the compounds and their mode of action (Figure 1). The first class is a single-ligand molecule that directly interacts with the target protein to induce degradation. This class of molecules include fulvestrant, a selective estrogen receptor downregulator (SERD) against estrogen receptor-α (ERα) (Osborne et al., 2004) which is approved in the clinic against breast cancers expressing ERα, and a selective androgen receptor downregulator (SARD) against androgen receptor (AR) (Omlin et al., 2015) currently under clinical evaluation. These downregulators are likely to recapitulate the degradation mechanism reported as hydrophobic tagging (Neklesa et al., 2011). Another example in this class is inhibitor of apoptosis protein (IAP) antagonists (Fulda and Vucic, 2012) that induce degradation of cIAP1/2, and some compounds are under clinical development. In addition, Boc3Arg-linked ligands that localize target proteins directly to the 20S proteasome are also grouped in this class (Shi et al., 2016). Thus, molecules in this class can effectively induce degradation of target proteins; however, the number of the proteins targeted for degradation is limited.

The second class of molecules is the single-ligand molecules that interact with E3 ubiquitin ligases to modulate substrate selectivity. This class of molecules is known as E3 modulators and molecular glues. Thalidomide was the first E3 modulator identified and interacts with CRBN (Ito et al., 2010), a substrate recognition subunit of the Cullin-RING-ubiquitin ligase (CRL) complex. Thalidomide and an analog lenalidomide induce the degradation of transcription factors Ikaros and Aiolos (Krönke et al., 2014; Lu et al., 2014). Modification of the side chain of thalidomide alters substrate selectivity, and lenalidomide and CC-885 induce ubiquitylation and degradation of casein kinase 1α (CK1α) (Krönke et al., 2015) and a translation termination factor GSPT1 (Matyskiela et al., 2016), respectively. Sulfonamides such as Indisulam and E7820 are reported to interact with DCAF15, another substrate recognition subunit of the CRL complex, and induce the ubiquitylation and degradation of a splicing factor RBM39/CAPERα (Han et al., 2017; Uehara et al., 2017). Plant hormones including auxin and gibberellin are also categorized in this class. Auxin interacts with F-box proteins TIR1 and AFB2 in the SCF ubiquitin ligase complex, and recruits a transcriptional repressor to be ubiquitylated and degraded by the proteasome, which in turn activates the expression of auxin-responsive genes (Dharmasiri et al., 2005; Kepinski and Leyser, 2005).

The third class is a chimeric molecule, where an E3 ligand and a target ligand are conjugated to form one molecule. This class of molecules was developed under different names such as PROTACs and SNIPERs, but they are designed to crosslink the target protein and an E3 ubiquitin ligase to induce the degradation of the target protein, and therefore, their mode of action is almost identical.

## Development of Chimeric Degrader Molecules

The first PROTAC reported came from the laboratories of Crews and Deshaies by using a peptide sequence recognized by an F-box protein β-TRCP to recruit the E3 ubiquitin ligase complex involving β-TRCP (Sakamoto et al., 2001). This PROTAC induces ubiquitylation and degradation of a target protein MetAP-2 in an *in vitro* cell-free system, but cannot penetrate into cells efficiently. In collaboration with Ciulli, Crews et al. developed small molecule ligands for VHL (Buckley et al., 2012a,b), and developed small molecule PROTACs (Bondeson et al., 2015; Buckley et al., 2015). These PROTACs induce degradation of various target proteins at nanomolar or sub-nanomolar concentrations in cell culture systems and induce the degradation of target proteins in *in vivo* xenograft models.

We have studied IAP family proteins that are frequently overexpressed in cancer cells and found that a small molecule methyl bestatin (MeBS) induces auto-ubiquitylation and proteasomal degradation of cIAP1 (Sekine et al., 2008). By using MeBS as a ligand for cIAP1, we developed the first SNIPER that induced the degradation of cellular retinoic acid binding protein II (CRABP2) (Itoh et al., 2010). The activity of SNIPERs was then markedly improved by adopting high affinity ligands for IAPs, and the improved SNIPERs at nanomolar concentrations effectively induced degradation of target proteins by recruiting XIAP and cIAP1 (Ohoka et al., 2017, 2018). Some of the SNIPERs were demonstrated to induce degradation of target proteins in an *in vivo* xenograft model, which results in antitumor activity.

**Figure 2:**
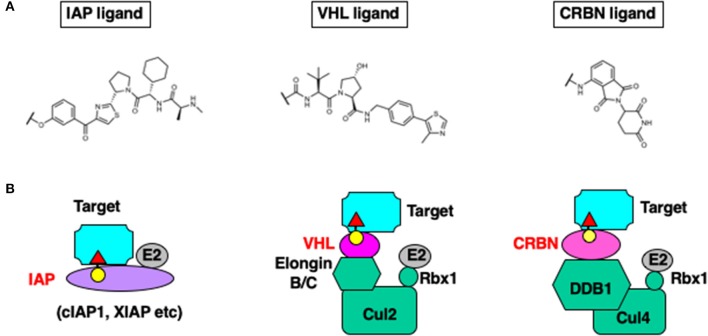
Chemical structure of the E3 ligands **(A)**, and the E3 ligase complexes hijacked by chimeric degraders **(B)**.

Handa et al. reported that CRBN is the direct target of thalidomide that has teratogenic activity (Ito et al., 2010). Bradner et al. then developed another family of chimeric molecules containing thalidomide as a ligand for CRBN that induce degradation of bromo domain proteins (Winter et al., 2015). The thalidomide-based chimeric molecules also induce degradation of target proteins at nanomolar concentrations and show activity in an *in vivo* xenograft model. Figure 2 illustrates the E3 ligands and ubiquitin ligase complexes recruited to target proteins.

## Features of the Chimeric Degrader Molecules

Because of the modular structure of chimeric degrader molecules, it is possible to rationally design and develop a novel degrader molecule against a protein of interest by substituting the target ligand. The target ligand does not need to inhibit the activity of the target protein, and therefore, a poor inhibitor that has insufficient activity to inhibit the target protein can be converted to a potent degrader when incorporated into chimeric degrader molecules. Theoretically, a ligand that interacts with any domain of the target protein can effectively capture the target to induce degradation. A higher binding affinity of the target ligand is preferable (Ohoka et al., 2018); however, some target proteins cooperatively interact with E3 ligases in the presence of chimeric molecules (Gadd et al., 2017), implying that low affinity ligands can also be used to develop potent chimeric degraders.

There are only a few E3 ligases among the more than 600 E3 ligases in cells that can currently be successfully recruited to target proteins for degradation. It should be noted that recruiting different E3 ubiquitin ligases to the same target protein results in different degradation potencies (Lai et al., 2016; Shibata et al., 2018), suggesting that finding the best combination of target protein and E3 ligase is important in the development of potent degraders. In this context, it is important to expand the repertoire of E3 ligands to recruit a wide variety of E3 ligases to target proteins. Some of the E3 ubiquitin ligases are expressed in a tissue specific and tumor specific manner. If such an E3 ligase can be recruited to target proteins, we anticipate that degradation of target proteins will be restricted to a tissue type or only tumor cells, which could be more advantageous in terms of selective toxicity. The number of E3 ligands is gradually increasing (Lu et al., 2018; Spradlin et al., 2019; Ward et al., 2019; Zhang X. et al., 2019) but they require improvement to induce degradation at lower concentrations. Recently, cells resistant against PROTACs have been reported (Zhang L. et al., 2019), and the resistance mechanism resides in the alteration of the ubiquitylation machinery rather than the target proteins. To overcome such resistance, it is possible to recruit different E3 ubiquitin ligases to restore the degradation of the target proteins, which further accentuates the importance of developing novel E3 ligands.

## Chimeric Degrader Molecules as Probes to Understand the Ubiquitin Code

Although ubiquitin was originally identified as an essential factor to induce proteasomal degradation of many proteins, it is widely accepted that ubiquitin plays a role in a variety of cellular phenomena, such as internalization of membrane proteins, autophagy, DNA repair, and signal transduction. The diversity in the linkage and modification of the ubiquitin chain, which is called the ubiquitin code, is assumed to be recognized by different decoder molecules that may mediate different cellular responses (Komander and Rape, 2012). To understand the ubiquitin code in more detail, it would be useful to write a ubiquitin code by chimeric molecules recruiting different E3 ubiquitin ligases to determine whether different cellular responses could be induced by different ubiquitin codes encrypted by various E3 ubiquitin ligases. For this purpose, ubiquitylation of tagged-proteins with chimeric degraders could provide a comprehensive system to ubiquitylate a variety of target proteins (Neklesa et al., 2011; Natsume et al., 2016; Hattori et al., 2017; Nabet et al., 2018; Okitsu et al., 2018).

## Conclusion

Technologies to induce targeted protein degradation have been established recently. These technologies are useful for developing novel drugs, and have promoted a number of drug development research programs by pharmaceutical companies, bio-ventures, and academia. The results of the first clinical phase I studies of PROTACs (ARV-110 against AR and ARV-471 against ER) were released recently demonstrating acceptable safety profiles. However, these technologies are still in their infancy and have significant room for improvement. These technologies should be further refined, and ultimately applied to clinical drug development as well as basic research to understand the ubiquitin biology.

## Author Contributions

MN, NO, NS, and YT wrote and checked the manuscript.

### Conflict of Interest

MN received a research fund from Daiichi Sankyo Pharmaceutical Co., Ltd. The remaining authors declare that the research was conducted in the absence of any commercial or financial relationships that could be construed as a potential conflict of interest.
